# Motor resonance in left- and right-handers: evidence for effector-independent motor representations

**DOI:** 10.3389/fnhum.2013.00033

**Published:** 2013-02-13

**Authors:** Luisa Sartori, Chiara Begliomini, Umberto Castiello

**Affiliations:** Dipartimento di Psicologia Generale, Università degli Studi di PadovaPadova, Italy

**Keywords:** motor representations, handedness, action observation, motor resonance, transcranial magnetic stimulation, motor evoked potentials

## Abstract

The idea of motor resonance was born at the time that it was demonstrated that cortical and spinal pathways of the motor system are specifically activated during both action-observation and execution. What is not known is if the human action observation-execution matching system simulates actions through motor representations specifically attuned to the laterality of the observed effectors (i.e., effector-dependent representations) or through abstract motor representations unconnected to the observed effector (i.e., effector-independent representations). To answer that question we need to know how the information necessary for motor resonance is represented or integrated within the representation of an effector. Transcranial magnetic stimulation (TMS)-induced motor evoked potentials (MEPs) were thus recorded from the dominant and non-dominant hands of left- and right-handed participants while they observed a left- or a right-handed model grasping an object. The anatomical correspondence between the effector being observed and the observer's effector classically reported in the literature was confirmed by the MEP response in the dominant hand of participants observing models with their same hand preference. This effect was found in both left- as well as in right-handers. When a broader spectrum of options, such as actions performed by a model with a different hand preference, was instead considered, that correspondence disappeared. Motor resonance was noted in the observer's dominant effector regardless of the laterality of the hand being observed. This would indicate that there is a more sophisticated mechanism which works to convert someone else's pattern of movement into the observer's optimal motor commands and that effector-independent representations specifically modulate motor resonance.

## Introduction

The general ability to achieve a goal by means of different effectors suggests that an abstract movement representation is activated regardless of the specific muscle involved (Lashley, 1930). Evidence for effector-independent motor representations has been obtained from studies evaluating the influence of learning a task with one effector on performance with another (Grafton et al., 1998) or showing how covert and overt imitation are goal-directed (Bekkering et al., 2000; Campione and Gentilucci, 2010). Much less is known about the characteristics of motor representations implemented within the action observation-execution matching system implying that perceiving another person's body movements activates corresponding motor representations in the observer's brain (Gallese et al., 1996; Prinz, 1997). Termed motor resonance, this process explains a number of phenomena such as motor contagion (Bouquet et al., 2011), unintentional imitation (Chartrand and Bargh, 1999), motor interference (Kilner et al., 2003; Blakemore and Frith, 2005; Gowen et al., 2008), automatic imitation (Knuf et al., 2001; Wilson and Knoblich, 2005; Heyes, 2011), and action understanding (Iacoboni et al., 1999; Buccino et al., 2004; Rizzolatti and Craighero, 2004).

An aspect concerned with motor resonance which remains partially unsolved is how the laterality of an observed effector shapes a motor resonant response. Investigating this issue would help to clarify whether motor representations developed during action observation are effector-dependent or -independent. Preliminary data have shown that each hemisphere is activated to a greater extent when a person is viewing actions conducted by the contralateral hand, a finding congruent with the pattern of motor representation in each hemisphere (Aziz-Zadeh et al., 2002). More recent evidence has indicated that observation of very simple one-hand movements evokes a bimanual resonant response (Borroni et al., 2008), suggesting that motor resonance does not encode the laterality of the observed hand but a more abstract representation of the movement. Another study reports that left- and right-handers differ in the degree of lateralization and involvement of the action observation/execution matching system during action production and action observation (Rocca et al., 2008; see also Rocca and Filippi, 2010). During execution, left-handers showed a more bilateral pattern of activation in areas of the motor system including the inferior frontal gyrus. During observation, left-handers showed an increased involvement of the superior temporal sulcus. Such differences in activation were interpreted as due to an increased involvement of imitative processes during execution and observation in left-handers as compared to right-handers. However, by adopting a more fine-grained analysis strategy to investigate the issue of laterality during action production as during action observation, Willems and Hagoort (2009) were unable to find selective differences in left- and right-handers depending on modality (execution vs. observation). They showed that neural differences related to preferred handedness during action production were also present during observation of the same action in several parts of the motor system.

From the above mentioned evidence it is evident that the influence of an observer's hand preference on the motor resonant response continues to be debated. Since motor resonance is usually studied in the dominant hand of right-handed participants who are observing right-handed models, it is not clear to what extent previously reported effects reflect a spontaneous manual preference toward the right effector. As left-handed participants have often been excluded from studies in the past, our understanding of the relationship between motor resonance and motor dominance is, in effect, quite limited. If we want to examine motor representation in a more discriminating way, we need to understand the pattern of motor resonance in left-handed subjects. Just as we need to know how the motor system resonates when the observed effector does not correspond to the observer's dominant effector. The present study has attempted to answer these questions.

We used Transcranial magnetic stimulation (TMS) to monitor alterations in corticospinal excitability (CS) that specifically accompany action observation by measuring the amplitude of motor evoked potentials (MEPs) elicited by single-pulse TMS (Fadiga et al., 1995; Strafella and Paus, 2000; Gangitano et al., 2001; Borroni et al., 2005; Montagna et al., 2005; Urgesi et al., 2006; Avenanti et al., 2007; Aglioti et al., 2008). MEPs were thus recorded from the abductor digiti minimi (ADM) muscle of the dominant and non-dominant hands of right- and left-handed participants as they watched video-clips. Half of the clips showed a model reaching and grasping an object with her right hand; the other half displayed the same model performing the same action with her left hand. The MEPs were recorded from the ADM muscle (i.e., the muscle serving little finger abduction) due to its involvement in whole-hand grasping.

We hypothesized that if motor representation is effector-dependent, then motor resonance should be guided only by an anatomical one-to-one correspondence between the effector of the model being observed and the participant's effector. Conversely, if motor representations promote an abstract effector-independent encoding of movements, then the process of motor simulation should not be limited to a direct matching between the model's and the participants' effectors. Motor resonance could occur in effectors different from the ones being observed if another person's actions are encoded at an abstract level.

## Materials and methods

### Participants

Thirty right-handed (16 females and 14 males, mean age 24 years, range 19–56) and 30 left-handed (24 females and 6 males, mean age 23 years, range 20–47) participants took part in the experiment. All had normal or corrected-to-normal visual acuity, and none had any contraindications to TMS (Wassermann, 1998; Rossi et al., 2009). The participants' degree of handedness was evaluated using a modified version of the Edinburgh Inventory (EHI) (Oldfield, 1971; Salmaso and Longoni, 1985). We converted the EHI total score into a dichotomous variable by computing the laterality quotient (LQ) that ranges from −100 (strong left handedness) to +100 (strong right-handedness), through the following standard expression: LQ = (R − L)/(R + L) × 100. R and L represent the total number of right- and left-hand items endorsed, respectively. A score below 0 (included) identified left-handed participants, while LQ > 0 detected right-handed participants. The LQ ranged between −100 and −14 (mean −63) for the left-handed participants. For the right-handed participants, it ranged between 67 and 100 (mean 89). The study protocol was approved by the Ethics Committee of the University of Padova and was carried out in accordance with the principles of the Declaration of Helsinki. All the participants gave their written informed consent to participate in the study before the experiments were conducted. While they were unaware of its purpose, the participants were partially debriefed once the experimental session was concluded. None of the participants experienced discomfort or adverse effects during the experiment.

### Experimental stimuli

To create the stimulus material, a model was filmed from an allocentric point of view naturally reaching and grasping a thermos with a whole hand grasp (WHG; i.e., the opposition of the thumb with the other fingers) using her right hand. The video-clip was then reflected on a horizontal plane using video editing procedures so that the model appeared to be reaching and grasping the same object with her left hand. An animation effect was obtained by presenting a series of 45 frames each lasting 33 ms (resolution 720 × 576 pixels, color depth 24 bits, frame rate 30 fps) plus the first and last frames which lasted 500 and 1000 ms, respectively.

### TMS stimulation and MEP recording

TMS was delivered using a 70-mm figure-of-eight coil connected to a Magstim 200^2^ stimulator (Magstim, Whitlan, Dyfed, Wales, UK). The coil was angled 45° relative to the interhemispheric fissure and perpendicularly to the central sulcus with the handle pointing laterally and caudally (Brasil-Neto et al., 1992; Mills et al., 1992). This orientation induces a posterior-anterior current in the brain which tends to activate corticospinal neurons indirectly via excitatory synaptic inputs (Di Lazzaro et al., 1998). TMS pulses were delivered over the left and right primary motor cortex (M1) areas corresponding to the hand region in two separate blocks (“left M1” and “right M1” blocks, respectively). The coil was positioned in correspondence with the optimal scalp position, defined as the position at which TMS pulses of slightly suprathreshold intensity consistently produced the largest MEP from the ADM muscle. The coil was held by a tripod and continuously checked by the experimenters to maintain consistent positioning. The individual resting motor threshold (rMT) was determined for each participant as the minimum intensity that induced reliable MEPs (≥50 μV peak-to-peak amplitude) in the relaxed muscle of the dominant hand in five out of ten consecutive trials (Rossini et al., 1994). The same stimulation intensity (110% of the rMT) was used for the left and right M1 sessions in each subject. Stimulation intensity during the recording session ranged between 40 and 65% of the maximum stimulator output intensity (mean 53%) for the right-handed participants. For the left-handed participants, it ranged between 39 and 61% of the maximum stimulator output intensity (mean 54%). Since each hemisphere is specialized in representing movements of the contralateral hand, MEPs were recorded from electrodes placed over the contralateral ADM. Electromyographic (EMG) recordings were made through pairs of 9 mm diameter Ag-AgCl surface electrodes. The active electrode was placed over the belly of the right ADM and the reference electrode over the ipsilateral proximal interphalangeal joint (belly-tendon montage). The electrodes were connected to an isolated portable ExG input box linked to the main EMG amplifier for signal transmission via twin fiber optic cable (Professional BrainAmp ExG MR, Brain Products, Munich, Germany). The ground electrode was placed over the participants' ipsilateral wrist and connected to the common input of the ExG input box. The raw myographic signals were bandpass filtered (20 Hz–1 kHz), amplified prior to being digitized (5 kHz sampling rate), and stored in a database for off-line analysis. Trials in which any EMG activity greater than 100 μV was present in the 100 ms window preceding the TMS pulse were discarded to prevent contamination of MEP measurements by background EMG activity. EMG data were collected for 200 ms after the TMS pulses were delivered.

### Procedure

Each participant was tested during a single experimental session lasting approximately 40 min. Testing was carried out in a sound-attenuated Faraday room. Each participant was seated in a comfortable armchair with his/her head positioned on a fixed head rest so that the eye–screen distance was 80 cm. Both arms were positioned on full-arm supports. Each participant was instructed to keep his/her hands in a prone position and as still and relaxed as possible.

The task was to pay attention to the visual stimuli presented on a 19″ monitor (resolution 1280 × 1024 pixels, refresh frequency 75 Hz, background luminance of 0.5 cd/m^2^) set at eye level. The participants were instructed to passively watch the video-clips and to avoid making any movements. In order to keep the participants fully attentive to what was being shown, they were told that they would be questioned at the end of the session about the visual stimuli presented.

During the “left M1” blocks, TMS-induced MEPs were acquired from the participant's right ADM muscle during stimulation of the left M1. During the “right M1” blocks, MEPs were acquired from the participant's left ADM muscle during stimulation of the right M1. The order in which the two blocks were delivered was counterbalanced across participants. Sixteen TMS-induced MEPs were acquired for each of the two blocks at the time the model's hand reached its maximum aperture just before contacting the object (35° frame), for a total of 32 MEPs per participant.

Prior to the video presentation, a baseline CS was assessed by acquiring 10 MEPs per block while the participants passively watched a white fixation cross on the black background on the computer screen. Ten more MEPs were recorded at the end of each block. By comparing the MEP amplitudes for the two baseline series it was possible to check for any CS changes related to TMS *per se* in each block. The average amplitude of the two series was utilized to set each participant's individual baseline for the data normalization process.

All the participants watched two types of video-clips presented in random order: the “right-hand” video in which a right-handed model performed a WHG to handle a thermos, and the “left-hand” video in which the model was seen reaching and grasping the same object with her left hand.

Each video presentation was followed by a 10 s rest interval. During the first 5 s of the rest period, a message reminding the participants to keep their hands still and fully relaxed appeared on the screen. A fixation cross was presented for the remaining 5 s. Stimuli presentation and the timing of TMS stimulation were managed by E-Prime V2.0 software (Psychology Software Tools Inc., Pittsburgh, PA, USA) running on a PC.

### Data analysis

For each condition, peak-to-peak MEP amplitudes recorded from the ADM muscle were measured and averaged. Those amplitudes deviating more than two standard deviations from the mean for each type of action and trials contaminated by muscular pre-activation were excluded as outliers (<3%). A paired-sample *t*-test (2-tailed) was used to compare the amplitude of MEPs recorded during the two series of baseline trials at the beginning and at the end of each block. Ratios were then computed using the participants' individual mean MEP amplitude recorded during the two fixation periods as baseline (MEP ratio = MEPobtained/MEPbaseline). In order to test any difference for the dominant and non-dominant hands in each subject and the LQ scores across the two groups, we performed a paired-sample *t*-test (2-tailed) on the mean baseline values of each hand in each subject and another *t*-test on the absolute score values of the LQ across the two groups. A mixed-design analysis of variance (ANOVA) was conducted on the MEP ratios with “model” (right-handed, left-handed) and “stimulated muscle” (left ADM, right ADM) as within-subjects factors and “group” (right-handed, left-handed) as between-subjects factor. Sphericity of the data was verified prior to performing statistical analysis (Mauchly's test, *p* > 0.05). *Post-hoc* pairwise comparisons were carried out using *t*-tests and the Bonferroni correction was applied for multiple comparisons. The comparisons between normalized MEP amplitude and baseline were performed using one-sample *t*-tests.

## Results

**Figure 1:**
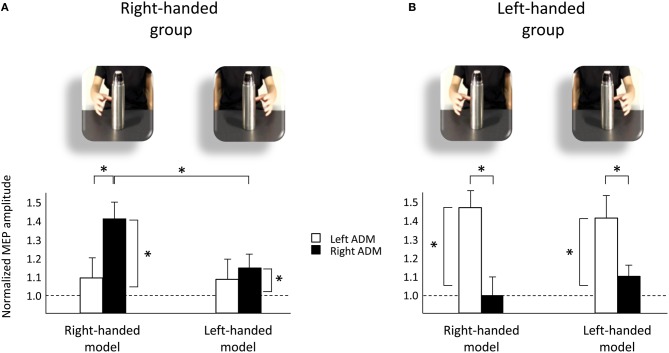
**Upper panels represent frames extracted from the two video-clips at the time-points at which TMS pulses were delivered.** Lower panels represent normalized MEP amplitude for left ADM (white bars) and right ADM (black bars) muscles across conditions (right-handed model, left-handed model) for right-handed **(A)** and left-handed **(B)** groups. Asterisks indicate significant comparisons (*p* < 0.05). Bars represent the standard error of means. Horizontal dotted lines indicate MEP baseline values.

The mean raw MEP amplitudes recorded during the two baseline series at the beginning and the end of each block were not significantly different in the right-handed participants neither during the “left M1” block (1138.85 vs. 999.37 μV, respectively; *t*_29_ = 0.62, *p* = 0.54) nor the “right M1” block (1048.82 vs. 851.08 μV, respectively; *t*_29_ = 0.92, *p* = 0.36). Similarly, the two baseline series were not significantly different in the left-handed participants neither during the “left M1” block (1389.83 vs. 1492.11 μV, respectively; *t*_29_ = −0.45, *p* = 0.66) nor the “right M1” block (1036.92 vs. 840.51 μV, respectively; *t*_29_ = 1.96, *p* = 0.06). This suggests that TMS *per se* did not induce any changes in CS during our experimental procedure. The absolute LQ score values in left-handers were significantly lower than in right-handers (63 vs. 89; *t*_11_ = −2.72, *p* = 0.02). This suggest that LQ during action execution was less lateralized in the left-hand group than in the right-hand group. Accordingly, a significant difference in the mean baseline values of the dominant and non-dominant hand was found in left handers (94.77 vs. 1455.85 μV, respectively; *t*_29_ = −2.31, *p* = 0.28), but not in right-handers (1065.73 vs. 66.75 μV, respectively; *t*_29_ = 0.71, *p* = 0.48). However, a non-significant correlation between the LQ and motor facilitation [(same hand preference) − (different hand preference)] in the dominant hand of both right-handers (Pearson's *r* = 0.953, *p* = 0.95) and left-handers (Pearson's *r* = 0.08, *p* = 0.19) seem to rule out the hypothesis of a strict correspondence between the LQ during action execution and action observation. The mean MEP ratios from the left and right ADM muscles for each model condition (right-handed, left-handed) are outlined in Figure 1. The mixed-design ANOVA on the normalized MEP amplitudes showed a significant “muscle by group” interaction [*F*_(1, 118)_ = 9.91, *p* < 0.005, η^2^_*p*_ = 0.15] and a significant “muscle by model by group” interaction [*F*_(1, 118)_ = 6.33, *p* < 0.05, η^2^_*p*_ = 0.10]. The results obtained for *post-hoc* contrasts are reported as follows.

### Effects of motor resonance

*Post-hoc* comparisons revealed statistically significant differences in the hand muscles of both groups. In particular, the MEP amplitudes for the right ADM muscle was greater than for the left one when the right-handed group observed the right-handed model (*p* < 0.05; Figure 1A). And the MEP amplitudes for the left ADM muscle were greater than that for the right one when the left-handed group was observing the left-handed model (*p* < 0.05; Figure 1B). This signifies that each group was resonating with their dominant hand as they observed models with their corresponding hand preference.

### Beyond motor resonance

*Post-hoc* comparisons for the left-handed group revealed that MEP activity was greater for the left than for the right ADM muscle also when they observed the right-handed model (*p* < 0.05; Figure 1B). Moreover, when CS activity for the dominant hand muscles was compared against baseline values, a statistically significant increase was found in MEP amplitudes of both groups regardless of the observer's and the model's hand preference (*p*_s_ < 0.05; Figures 1A,B). When the non-dominant hand muscles were assessed, instead, there was no statistically significant activation with respect to the baseline value for any condition (*p*_s_ > 0.05). The fact that there was a statistically significant activation in the dominant hand muscles of both right- and left-handers seems to suggest that observing another person's action leads to a generalized, no-specific effect in the dominant hand of both right- and left-handers and no effect on the non-dominant hand.

### Effects of observer's handedness

*Post-hoc* comparisons for the right-handed group revealed statistically significant differences across types of video. In particular, the MEP amplitudes for the dominant (right) ADM muscle were greater while they watched the right-handed with respect to the left-handed model (*p* < 0.05; Figure 1A). On the contrary, the MEP amplitudes for the dominant ADM muscle of the left-handed participants were not statistically different while they were observing the right- and the left-handed models (*p* > 0.05; Figure 1B).

### Effects of model's handedness

*Post-hoc* comparisons revealed statistically significant differences across groups. In particular, the MEP amplitudes for the right ADM muscle were greater in the right than in the left-handed group while they observed the right-handed model (1.40 vs. 1.03, respectively; *p* < 0.05; see also Figure 1). The MEP amplitudes for the left ADM muscle were greater for the left than for the right-handed participants both while they observed the right-handed model (1.48 vs. 1.11, respectively; *p* < 0.05; see also Figure 1) and the left-handed model (1.43 vs. 1.13, respectively; *p* < 0.05; see also Figure 1).

## Discussion

Are motor representations elicited during action observation specifically attuned to the laterality of the observed effectors (i.e., effector-dependent representations) or do these provide an abstract code of motor information (i.e., effector-independent representations)? Are motor resonance effects linked in some way to motor dominance? These are the questions that were addressed by our study.

The importance *(supremacy*) of an observer's hand preference in determining the pattern of CS regardless of the laterality of the effector being observed has been demonstrated here for the first time. The anatomical correspondence between the hand being observed and the hand belonging to an observer classically reported in the literature was confirmed in right-handers only when MEPs from the dominant hand of participants observing models with their same hand preference were recorded (Fadiga et al., 1995; Strafella and Paus, 2000; Gangitano et al., 2001; Borroni et al., 2005; Montagna et al., 2005; Urgesi et al., 2006; Avenanti et al., 2007; Aglioti et al., 2008). This correspondence extended to left-handers but, independently from handedness, motor resonance disappeared when the non-dominant hand was considered. Consistent with the idea of an effector-independent representation, when they observed models with a different hand preference, both left- and right-handers showed motor resonance effects in their dominant hand. Though to a lesser degree in right-handers, who showed a greater amplitude in their dominant hand when they were observing a right- with respect to a left-handed model. These findings confirm and extend previous literature on the effect of preferred handedness during action observation (Borroni et al., 2008; Rocca et al., 2008; Willems and Hagoort, 2009; Rocca and Filippi, 2010) by revealing a stronger lateralized motor resonance in right-handers with respect to left-handers. In first instance the analysis of the interaction between handedness and model's hand might confirm a complex interplay between areas part of the action observation/execution matching system as previously demonstrated by neuroimaging investigations (Rocca et al., 2008). Furthermore, the fact that left-handers seem to equally translate any observed motor program into their dominant effector concords with evidence of more bilaterally spread brain functions in left- than in right-handers (Matsuo et al., 2002; Jorgens et al., 2007; Krombholz, 2008; Müller et al., 2011). In particular, Cabinio and colleagues (2010) showed that activation of the parieto-frontal circuit of the action observation-execution matching system evoked by observation of grasping actions is strongly lateralized in right-handers. In left-handers, on the other hand, the pattern of cortical activation is less lateralized. It is possible that living in a “right-handed world” has modified the tuning of the action observation-execution matching system, therefore hindering left-handers from fully lateralizing their manual preference and increasing the natural disposition of right-handers toward right-handed actions. The present findings suggest that left-handers might be able to deal with this “right” world essentially by resonating with right-handers. A recent fMRI study suggested that a predominant activity in the left parietal cortex would be at the basis of the effector-independent encoding of movement (Swinnen et al., 2010).

An alternative explanation for the facilitation found in the dominant hand of participants observing models with a different hand preference could be found in the general effect of specular imitation, which is a special case of spatial stimulus-response compatibility (SRC, Brebner et al., 1972). The SRC theory sustains that a compatible mapping of stimulus and response leads to faster responses with respect to an incompatible mapping. Previous studies have suggested that spatial compatibility is an important mechanism underlying imitation (van Schie et al., 2008; Catmur and Heyes, 2011; Mengotti et al., 2013). In the present study, the model's right hand was indeed specular with respect to the participant's left hand and vice versa. As a consequence, the hands of the observed model and of the observer shared the same spatial finger position, and this could explain the facilitation that was noted. This explanation, however, does not clarify the lack of facilitation for the non-dominant hand nor does it explicate the results concerning the dominant hand of the participants observing models with a similar hand preference. Although spatial compatibility is certainly an important element which modulates action imitation, our findings indicate that it is probably not the only factor to do so. It must be remembered, in any case, that the participants in our study were not directed to perform actions, but to passively observe. In this respect, it is difficult to compare our findings with previous results detected during imitation tasks (thus not allowing to rule out the bias associated with task execution). *Action observation is another and different feature of the action observation/execution matching system*.

In view of the fact that motor resonance reflects the motor representation evoked by a perceived action in an observer, our results suggest that the perceptual-motor matching of an observed action is facilitated when an observer sees a movement performed by a model with the same hand preference. But they also support the hypothesis of a more sophisticated rather than a traditional direct-matching model of motor resonance.

The direct-matching hypothesis postulates that viewing an action automatically evokes in the observer a representation of the motor commands necessary to execute that same action. TMS experiments typically show that observed movements are processed in a strictly time-locked, muscle specific fashion (Baldissera et al., 2001; Gangitano et al., 2001; Borroni et al., 2005; Montagna et al., 2005; Borroni and Baldissera, 2008; Candidi et al., 2008; Alaerts et al., 2009; Cavallo et al., 2011). While it is unclear how the direct-matching hypothesis deals with handedness, the findings outlined here suggesting that the perceptual-motor mapping of a movement is also sensitive to the observer's handedness complement those studies and take research one step further.

Previous findings showing that motor resonance is very precise might seem at odds with the notion of a more abstract action representation. According to a recently proposed hypothesis (Lepage et al., 2010; Lago and Fernandez-del-Olmo, 2011), there are two different mechanisms governing motor resonance: the first maps an observed action in terms of its goal and the second specifies the muscles involved in that action. Both the action goal and the motor program are encoded during observation of action preparation, but the specific muscles involved in the action are likewise encoded at the moment that the hand-object interaction actually takes place. Data from our study are consistent with that hypothesis in view of the fact that MEPs were acquired before the contact phase was reached. It cannot be excluded that a more specific representation (i.e., reflecting the model's handedness) is activated during observation of the actual hand-object interaction.

Consistent with the hypothesis that there are two separate processes for action observation, some investigators have distinguished between low- and high-level resonance mechanisms (Rizzolatti et al., 2002). Low level motor resonance can be considered a basic mechanism mirroring phenomenon of direct matching between perception and action thought to be the basis for motor contagion and unintentional imitation (Chartrand and Bargh, 1999) while more complex forms of action understanding probably require resonance at higher functional levels. In particular, the capacity to recognize another person's intention, thus allowing action anticipation and permitting coordination with others, could reflect a higher cognitive level (Hurley, 2005). Interestingly, some studies on action observation have shown that there is a preference for the outcome of the action rather than for the actual hand kinematics involved (Bach et al., 2005; van Elk et al., 2008, 2011; Cattaneo et al., 2009). Conversely, other studies seem to suggest a direct coupling between visual aspects of an observed action and motor cortex excitability (Gangitano et al., 2001; Maeda et al., 2002; Alaerts et al., 2009; Cavallo et al., 2011, 2012). Altogether, these apparently contradictory findings are consistent with the hypothesis that there are two different levels of motor representations: one providing a literal copy of the observed action and the other involving higher cognitive aspects. Notably, in our study the observed action was seen from an allocentric perspective. That is, the viewpoint consistent with looking at someone else's hand performing an action. This perspective entails a complex transformation of the visual information to a body-centered motor frame of reference, and this probably requires a more sophisticated level of motor representation with respect to actions seen from an egocentric perspective. It would be very useful if abstract motor representations could functionally transfer motor resonance from the observed to the own's preferred hand. Shmuelof and Zohari (2008) have shown that observed actions are remapped in the superior parietal lobule to the hand that will probably be used to replicate the action toward the relevant object in space. This mapping occurs without imitation, providing further evidence for an automatic action-simulation system in the parietal cortex. As long as an object becomes relevant to the goal of an action, it is conceivable that a highly efficient mechanism enables subjects to correctly plan movements toward the same target in a functional action-specific mode. Highly efficient systems are needed in the face of the complex, dynamic environments in which humans move about in, often characterized by object-related actions.

The aim of the present study was to provide further information about the relations between motor representation, resonance, and dominance.

A neutral motor representation attuned to both right- and left-hands being observed seems then to be at work in left-handers and—to a lesser degree—in right-handers. Our results extend previous evidence, showing that the observer's handedness shapes motor resonance in right- as well as in left-handers regardless the identity of the observed hand. And that the correspondence between model's and observer's effector is no longer revealed in their non-dominant hand.

Assuming this modulation effect is an index of motor representations' capability of taking into account the observer's hand dominance, the findings outlined here can be considered evidence for a sophisticated mechanism which converts another person's pattern of movement into optimal motor commands in an observer.

These findings, finally, clarify an important aspect of the action observation-execution matching system, indicating that motor resonance is mediated by effector-independent motor representations.

### Conflict of interest statement

The authors declare that the research was conducted in the absence of any commercial or financial relationships that could be construed as a potential conflict of interest.
